# Combined transcriptomic and metabolomic analysis reveals a role for adenosine triphosphate-binding cassette transporters and cell wall remodeling in response to salt stress in strawberry

**DOI:** 10.3389/fpls.2022.996765

**Published:** 2022-09-06

**Authors:** Shuangtao Li, Linlin Chang, Rui Sun, Jing Dong, Chuanfei Zhong, Yongshun Gao, Hongli Zhang, Lingzhi Wei, Yongqing Wei, Yuntao Zhang, Guixia Wang, Jian Sun

**Affiliations:** ^1^Institute of Forestry and Pomology, Beijing Academy of Agriculture and Forestry Sciences, Beijing, China; ^2^Beijing Engineering Research Center for Strawberry, Beijing, China; ^3^Key Laboratory of Biology and Genetic Improvement of Horticultural Crops (North China), Ministry of Agriculture, Beijing, China

**Keywords:** ABC transporter, cell wall remodeling, salt stress, salinity tolerance, strawberry

## Abstract

Strawberry (*Fragaria* × *ananassa* Duch) are sensitive to salt stress, and breeding salt-tolerant strawberry cultivars is the primary method to develop resistance to increased soil salinization. However, the underlying molecular mechanisms mediating the response of strawberry to salinity stress remain largely unknown. This study evaluated the salinity tolerance of 24 strawberry varieties, and transcriptomic and metabolomic analysis were performed of ‘Sweet Charlie’ (salt-tolerant) and ‘Benihoppe’ (salt-sensitive) to explore salt tolerance mechanisms in strawberry. Compared with the control, we identified 3412 differentially expressed genes (DEGs) and 209 differentially accumulated metabolites (DAMs) in ‘Benihoppe,’ and 5102 DEGs and 230 DAMs in ‘Sweet Charlie.’ DEGs Gene Ontology (GO) enrichment analyses indicated that the DEGs in ‘Benihoppe’ were enriched for ion homeostasis related terms, while in ‘Sweet Charlie,’ terms related to cell wall remodeling were over-represented. DEGs related to ion homeostasis and cell wall remodeling exhibited differential expression patterns in ‘Benihoppe’ and ‘Sweet Charlie.’ In ‘Benihoppe,’ 21 ion homeostasis-related DEGs and 32 cell wall remodeling-related DEGs were upregulated, while 23 ion homeostasis-related DEGs and 138 cell wall remodeling-related DEGs were downregulated. In ‘Sweet Charlie,’ 72 ion homeostasis-related DEGs and 275 cell wall remodeling-related DEGs were upregulated, while 11 ion homeostasis-related DEGs and 20 cell wall remodeling-related DEGs were downregulated. Kyoto Encyclopedia of Genes and Genomes (KEGG) pathway analyses showed only four KEGG enriched pathways were shared between ‘Benihoppe’ and ‘Sweet Charlie,’ including flavonoid biosynthesis, phenylalanine metabolism, phenylpropanoid biosynthesis and ubiquinone, and other terpenoid-quinone biosynthesis. Integrating the results of transcriptomic and metabolomics analyses showed that adenosine triphosphate-binding cassette (ABC) transporters and flavonoid pathway genes might play important roles in the salt stress response in strawberry, and DAMs and DEGs related to ABC transporter and flavonoid pathways were differentially expressed or accumulated. The results of this study reveal that cell wall remodeling and ABC transporters contribute to the response to salt stress in strawberry, and that related genes showed differential expression patterns in varieties with different salt tolerances. These findings provide new insights into the underlying molecular mechanism of strawberry response to salt stress and suggest potential targets for the breeding of salt-tolerant strawberry varieties.

## Introduction

Soil salinization is a significant environmental factor threatening food production and food security. Currently, soil salinization affects 800 million hectares of farmland worldwide, and every year about 1–2% of available acreage for farming is compromised due to soil salinity (Etesami and Beattie, 2018; Bomle et al., 2021). Furthermore, with temperature and precipitation changes related to climate change, irrigation, increasing sea level, and the use of wastewater all increase agricultural soil salinization worldwide (Koohbor et al., 2019; Shah et al., 2022). Saline soils lead to osmotic, ionic, and oxidative stress in plants, affecting overall plant growth and yield (Arora et al., 2020; Bomle et al., 2021). Strawberry is highly sensitive to salt stress, and previous studies have shown that under increased salt stress, there is a decrease in plant biomass and chlorophyll content, and negative impacts on organic acids, soluble solids, and the physical appearance of fruits (Kaya et al., 2019). Generally, the yield loss and commodity fruit rate reduction caused by salt stress significantly reduce economic benefits (Keutgen and Pawelzik, 2007; Suarez and Grieve, 2013; Ferreira et al., 2019). However, mild salt stress has a positive impact on certain desirable metabolites such as anthocyanins and phenolic compounds, compounds that are involved in plant stress responses (Vanessa et al., 2016). The additional cost of a water purification system will significantly raise the cost of production, so the effective breeding of salt tolerant varieties is an important way to counteract soil salinization for the strawberry industry. The use of salt tolerant varieties such as ‘Albion,’ ‘San Andreas,’ and ‘Camarosa’ in a breeding program can improve the performance of offspring under saline conditions (Sun et al., 2015). In our breeding program, F1 offspring, ‘Jingtaoxiang’ (‘Darselect’ × ‘Akihime’) (Wang et al., 2018b) showed significantly higher salt tolerance compared to ‘Akihime,’ the salt sensitive parent. Thus, further study on the molecular mechanisms involved in the response of different strawberry varieties to salt stress is needed to provide a theoretical basis for breeding.

Flavonoids are major non-enzymatic scavengers of reactive oxygen species (ROS), with antioxidant capacities. These compounds play a major role when plants suffer from salt stress. The flavonoid biosynthetic genes *CHS*, *CHI*, and *F3H* function as positive regulators to modulate plant salt stress tolerance by increasing the accumulation of flavonoids (Mahajan and Yadav, 2014; Chen et al., 2015; Wang et al., 2018a; Jayaraman et al., 2021). AtROS1-mediated demethylation improves the expression levels of flavonoid biosynthesis and antioxidant-related genes to increase salt stress tolerance (Bharti et al., 2015). In rice, SQD2.1 acts in the glycosylation of flavonoids and improves the scavenging of ROS to improve salt stress tolerance (Zhan et al., 2019). *CrUGT87A1* from *Carex rigescens* is a positive regulator of plant salt tolerance, and can enhance plant antioxidation capability through increased flavonoid accumulation to improve salt tolerance in plants (Zhang et al., 2021b). The R2R3-MYB transcription factors are core regulators of flavonoid biosynthesis and can modulate plant salt stress tolerance by controlling flavonoid biosynthesis and accumulation (Li et al., 2019a; Wang et al., 2020, 2021).

At the cellular level, the cell wall is the first barrier for plants to respond to salt stresses. Decreased turgor pressure induced by salt stress restricts cell expansion and division and the cell wall responds to salt stress by resisting cell turgor changes (Zhao et al., 2021b). Components of the cell wall such as cellulose, lignin, and xyloglucan have been implicated in the salt tolerance response. OsCSLD4, a major regulator of cell wall polysaccharide synthesis, is involved in the response to salt stress in rice by affecting abscisic acid biosynthesis to regulate osmotic stress tolerance (Zhao et al., 2021a). Companion of Cellulose Synthase 1 (CC1) promotes plant growth under salt stress *via* control microtubule bundling and dynamics (Kesten et al., 2019). Xyloglucan endotransglucosylase-hydrolase 30 (XTH30) can alter cellulose synthesis and negatively affect salt tolerance (Yan et al., 2019). MdSND1 and AgNAC1 participate in the response to salt stress by regulating lignin biosynthesis or accumulation (Chen et al., 2020; Duan et al., 2020). Lignin biosynthesis and accumulation make a critical contribution to the adaptation of plants to high-salt stress (Chun et al., 2019), and the MdMYB46 transcription factor enhanced salt stress tolerance by activating lignin biosynthesis-related genes (Chen et al., 2019).

Significant progress has been made, but further studies to identify regulatory factors are needed to understand the underlying mechanisms of the strawberry salt stress response and to facilitate salt tolerant breeding efforts. In this study, the salt tolerances of 24 strawberry varieties were investigated with varying NaCl concentrations. Based on the salt damage indices, tested varieties were clustered as salt tolerant, salt sensitive, or salt hypersensitive. To explore the molecular mechanism of strawberry response to salt stress, two strawberry varieties with different salt sensitivity were used to perform transcriptomic and metabolomic analysis. We hypothesized the presence of common and distinct pathways in the response to salt stress in different varieties. To ensure we evaluated varieties with an intact salt response network, we selected a salt-sensitive variety instead of a salt-hypersensitive variety to unravel the mechanism of the strawberry response to salt stress. ‘Sweet Charlie’ (salt-tolerant) and ‘Benihoppe’ (salt-sensitive), the top two varieties with the largest planting area and the highest genetic contribution value in strawberry breeding in China (Chang et al., 2018), were selected in this study. The results further our understanding of the mechanism of the response of strawberry to salt stress and provide a theoretical basis for further study into regulation of salt stress tolerance in strawberry.

## Materials and methods

### Plant growth and salt stress treatment

In this study, 24 strawberry varieties from the National Strawberry Germplasm Repository (Beijing, China) were used to investigate salt tolerance under different concentrations of NaCl solution, which included four day-neutral varieties: ‘Albion,’ ‘San Andreas,’ ‘Portola,’ and ‘Monterey,’ and twenty short-day varieties: ‘Benihoppe,’ ‘Akihime,’ ‘Ssanta,’ ‘Tokun,’ ‘Kinuama,’ ‘Tochiotome,’ ‘Sweet Charlie,’ ‘Tianxiang,’ ‘Yanxiang,’ ‘Shuxiang,’ ‘Hongxiutianxiang,’ ‘Jingyixiang,’ ‘Jingchengxiang,’ ‘Jingquanxiang,’ ‘Jingzhangxiang,’ ‘Jingliuxiang,’ ‘Jingtaoxiang,’ ‘Pink Princess,’ ‘Snow White,’ and ‘Yanli,’ Strawberry seedlings were cultivated in 2.5-L containers with peat moss and perlite (2:1 v/v ratio) in a greenhouse with a 16-h photoperiod provided by supplemental lighting, 25/16°C (day/night) temperature cycle, 60% relative humidity, and 200 μmol m^–2^ s^–1^ light intensity. Salt stress treatments were conducted when the plants were at the 6–7 leaf stages. Plants were irrigated with four different concentrations of salt solutions: 50, 100, 150, or 200 mM NaCl for 50 days. To do this, 100 ml of the salt solution was applied once per week, and the same volume of fresh water was applied in the control treatment. Plants salt damage index (SDI) was measured as described by Zhong et al. (2021), and the SDI grades were scaled into six levels from 0 to 5, according to different damage symptoms observed in the leaves after salt treatment. The classification standard of plant salt damage was as follows: Grade 0: no symptoms of salt damage; Grade1: about 1/3 of the leaves showed wilting phenotype at leaf tips and leaf margins; Grade 2: about 1/2 of the leaves showed wilting and burn phenotype at leaf tips and leaf margins; Grade 3: about 2/3 of the leaves showed burn phenotype and the burn area was about 1/3; Grade 4: all leaves showed burn phenotype and the burn area was more than 1/2; Grade 5: all leaves were scorched. The SDI was counted as = Σ (salt damage series × number of plants with the corresponding salt damage level)/total number of plants tested. Biomass accumulation was measured as the dry weight of the whole plant. The experimental design included three randomized replicate blocks, with ten plants in each replicate block. The experiments were carried out in January, 2017 and January, 2018. To explore the molecular mechanism of strawberry response to salt stress, ‘Sweet Charlie’ and ‘Benihoppe’ were selected as strawberry varieties with different salt sensitivities and were subjected to transcriptomic and metabolomic analysis. ‘Sweet Charlie’ and ‘Benihoppe’ plants with 6–7 leaves were treated with 0 mM (control) or 100 mM NaCl solution, respectively. After 12 h of treatment, the young leaves were collected for ribonucleic acid (RNA) and metabolite extraction. After 10 days of treatment, the young leaves were collected for physiological indices determination.

### Measurement of physiological indices

To evaluate the effects of salt stress on physiological performance of ‘Sweet Charlie’ and ‘Benihoppe,’ biochemical indices of malondialdehyde (MDA), superoxide anion (H_2_O_2_ and O_2_^•–^) content, and superoxide dismutase (SOD), peroxidase (POD), and catalase (CAT) activities were measured. Each experiment was performed with three biological replicates.

To evaluate the degree of membrane lipid peroxidation, the content of MDA was measured as described by Feng et al. (2022). Briefly, 0.5-gram samples of leaves were ground in buffer solution, and then supernatants were mixed with equivalent volumes of thiobarbituric acid (TBA) and incubated at 100°C for 10 min. Finally, the supernatant absorbance was read at 450, 532, and 600 nm.

The content of H_2_O_2_ was detected using a hydrogen peroxide assay kit (Solarbio, China) (Wang et al., 2019). Briefly, 0.1-gram leaves were homogenized in l ml cold acetone, and then supernatants were mixed with 5% titanium sulfate. The resulting precipitates were dissolved in 2 M sulfuric acid and then the absorbance was read at 415 nm.

The concentration of O_2_^•–^ (superoxide anion) was determined using a superoxide anion assay kit (Solarbio, China) (Fang et al., 2021). Briefly, 0.1-gram leaves were ground in phosphate buffer solution, and then the supernatant was mixed with potassium phosphate buffer and hydroxylamine hydrochloride before reaction at 25°C for 20 min. Next p-aminobenzene sulfonic acid and 1-naphthylamine were added, the mixture was well-mixed and incubated at 30°C for 30 min, and then the absorbance was read at 530 nm.

To determine the activities of SOD, POD, and CAT, approximately 0.2-grams of leaves were ground and homogenized in sodium phosphate buffer and the resulting supernatants were assayed (Yu et al., 2021). SOD activity was determined according to the ability to inhibit the reduction of nitro blue tetrazolium (NBT) under light. POD activity was detected by measuring the oxidation of guaiacol, and CAT activity was measured by monitoring the consumption of hydrogen peroxide at 240 nm.

### Transcriptome sequencing and data analysis

Total RNA was extracted from leaves using TRIzol^®^ Reagent (Invitrogen, Carlsbad, CA, United States) following the manufacturer’s instructions. The quality and concentration of RNA were determined by an Agilent 2100 Bioanalyzer (Agilent, Palo Alto, CA, United States) and a DS-11 Spectrophotometer (DeNovix, Wilmington, DE, United States), respectively. RNA-seq transcriptome library was constructed using TruSeqTM RNA sample preparation Kit (Illumina, San Diego, CA, United States) following the instructions of the manufacturer. An RNA-seq sequencing library was sequenced on the Illumina HiSeq X Ten sequencing platform by using paired-end technology using three biological replicates for each treatment.

After removing and filtering low-quality sequences, the clean reads were separately aligned to the reference genome (*Fragaria* x *ananassa* Camarosa Genome v1.0) using TopHat software.^1^ The determination of fragments per kilobase of exon per million mapped reads (FRKM) was employed to calculate the expression level of each transcript. RSEM^2^ was used to quantify gene abundances. R statistical package software Empirical analysis of Digital Gene Expression in R (EdgeR)^3^ was utilized for differential expression analysis. *P*-value < 0.05 and |log_2_FC| > 1 were set as the threshold for significantly differential expression.

Functional-enrichment GO and KEGG analyses were performed to identify DEGs significantly enriched in GO terms and metabolic pathways at a Bonferroni-corrected *P*-value of ≤0.05 compared with the whole-transcriptome background. GO functional enrichment and KEGG pathway analyses were carried out using Goatools^4^ and KOBAS.^5^

### Metabolite profiling analysis

For the extraction of metabolites, fifty milligrams of strawberry leaves were mixed with 0.4 ml of an 80% aqueous methanol solution containing 0.02 mg/mL L-2-chlorophenylalanin as an internal standard. Chromatographic separation of the metabolites was performed on an UHPLC system (Thermo Fisher, Carlsbad, CA, United States) equipped with an ACQUITY UPLC HSS T3 (Waters, Milford, United States). The mass spectrometric data was collected using a Thermo UHPLC-Q Exactive HF-X Mass Spectrometer equipped with an ESI (electrospray ionization) source operating in either positive or negative ion mode. The metabolite profiling experiments were performed with six biological replicates for each treatment.

After UPLC-MS analyses, the raw data were imported into the Progenesis QI 2.3 (Non-linear Dynamics, Waters, United States) for peak detection and alignment. The preprocessing results generated a data matrix that was utilized for subsequent analyses. Human metabolome database^6^ (HMDB) and Metlin database^7^ were used for metabolite identification. Principal component analysis (PCA) and orthogonal partial least squares discriminate analysis (OPLS-DA) were performed on Majorbio Cloud Platform.^8^ Variable importance in the projection (VIP) were calculated in OPLS-DA model, and *p* values were estimated with paired Student’s *t*-test in single dimensional statistical analysis. Differentially accumulated metabolites (DAMs) between groups were selected with VIP ≥ 1, FC (fold change) ≥ 1 or FC ≤ 1 and *p* value ≤ 0.05. Differential metabolites between two groups were summarized and mapped into their biochemical pathways through metabolic enrichment and pathway analysis based on database search (KEGG^9^).

### Validation of quantitative real-time polymerase chain reaction differentially expressed genes

Quantitative real-time polymerase chain reaction (qPCR) was performed to validate the accuracy of the RNA-seq results. To do this, qPCR was performed on a 7500 Real-time PCR System (Applied Biosystems, Carlsbad, CA, United States), with the following cycling profile: 95°C for 20 s followed by 40 cycles at 95°C for 5 s, 60°C for 10 s, and 72°C for 20 s; followed by a melting curve. The relative gene expression levels were quantified by the 2^–ΔΔ*CT*^ method. *FaRPS1* (Merlaen et al., 2020) was selected as the internal control gene. The primer pairs are listed in Supplementary Table 1.

### Statistical analysis

Statistical analysis was conducted using SPSS 20.0 software, and statistical comparisons were performed using *t*-test in SPSS. Data are presented as the mean ± standard deviation (SD) values with at least three biological replicates.

## Results

### Evaluation of strawberry salt tolerance

**FIGURE 1:**
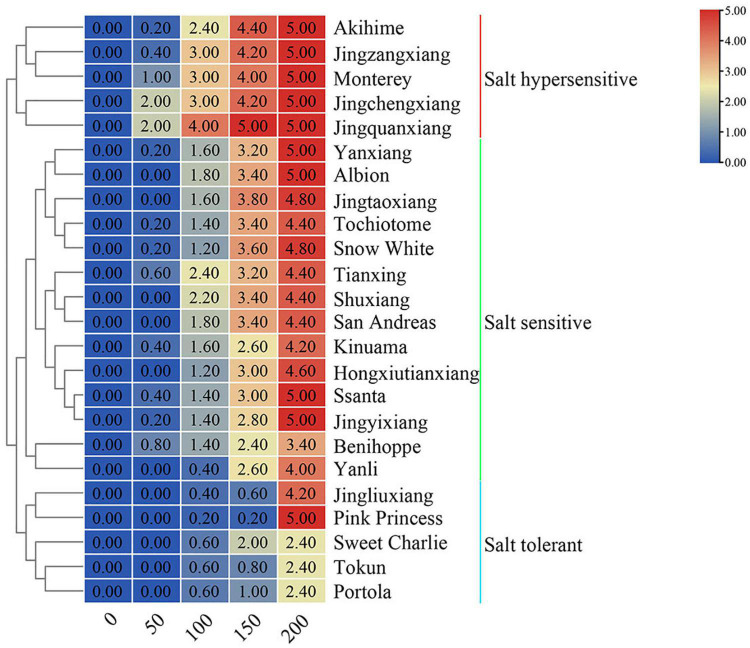
Salt damage index of 24 strawberry varieties under different concentration of NaCl. Strawberry plants with 6–7 leaves were irrigated with five different concentrations of salt solutions: 0, 50, 100, 150, or 200 mM NaCl for 50 days. The salt damage index grades were scaled into six levels from 0 to 5, according to different damage symptoms in the leaves after salt treatment. Based on the salt damage indices, tested varieties were clustered into three groups: salt tolerant, salt sensitive, and salt hypersensitive.

**FIGURE 2:**
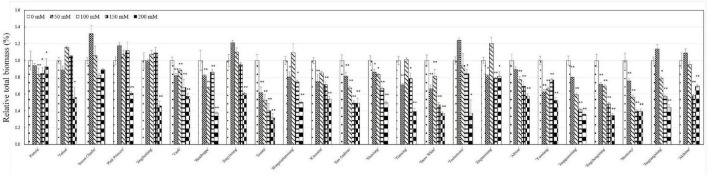
Relative total biomass of 24 strawberry varieties under different concentrations of NaCl. Strawberry plants with 6–7 leaves were irrigated with five different concentrations of salt solutions: 0, 50, 100, 150, or 200 mM NaCl for 50 days. Relative biomass accumulation was counted as the dry weight of the whole plant treated with different concentrations of salt solutions (50, 100, 150, or 200 mM) divided by the dry weight of the whole plant treated with fresh water. Data are means (±SD) of three independent experiments, *p < 0.05 or **p < 0.01.

With increasing salt concentration, all 24 strawberry varieties showed a salt injury phenotype with the salt injury index positively correlated with salt concentration. Based on the salt damage indices, tested varieties could be clustered into three groups: salt tolerant, salt sensitive, and salt hypersensitive (Figure 1). The five varieties with the highest salt tolerance were ‘Portola,’ ‘Tokun,’ ‘Sweet Charlie,’ ‘Jingliuxiang,’ and ‘Pink Princess.’ Three varieties were extremely salt tolerant, ‘Portola,’ ‘Tokun,’ and ‘Sweet Charlie,’ with the plants neither dying nor withering at the highest test concentration of 200 mM NaCl. The varieties ‘Akihime,’ ‘Jingzhangxiang,’ ‘Monterey,’ ‘Jingchengxiang,’ and ‘Jingquanxiang’ were salt hypersensitive, with these varieties showing moderate to a severe salt damage phenotype under the 100 mM NaCl treatment. The other 16 varieties were salt sensitive varieties, with biomass accumulation and growth and development obviously inhibited under salt stress. In this study, the total biomass of 24 strawberry varieties decreased with the increase of NaCl concentration, but there were differences among different varieties that fell into three classes (Figure 2). Compared with the control treatment, the biomass was slightly increased and then decreased with increasing NaCl concentration for ‘Tokun,’ ‘Sweet Charlie,’ ‘Pink Princess,’ ‘Jingliuxiang,’ ‘Jingyixiang,’ ‘Hongxiutianxiang,’ ‘Tochiotome,’ ‘Jingtaoxiang,’ ‘Jingzangxiang,’ and ‘Akihime’; the biomass of ‘Yanli,’ ‘Benihoppe,’ ‘Ssanta,’ ‘Kinuama,’ ‘San Andreas,’ ‘Shuxiang,’ ‘Tianxiang,’ ‘Snow White,’ ‘Albion,’ ‘Yanxiang,’ ‘Jingchengxiang,’ and ‘Monterey’ steadily decreased with increasing NaCl concentration, and there was little difference in the biomass of ‘Portola’ under different concentrations of NaCl.

### ‘Benihoppe’ is more sensitive to salt stress than ‘Sweet Charlie’

**FIGURE 3:**
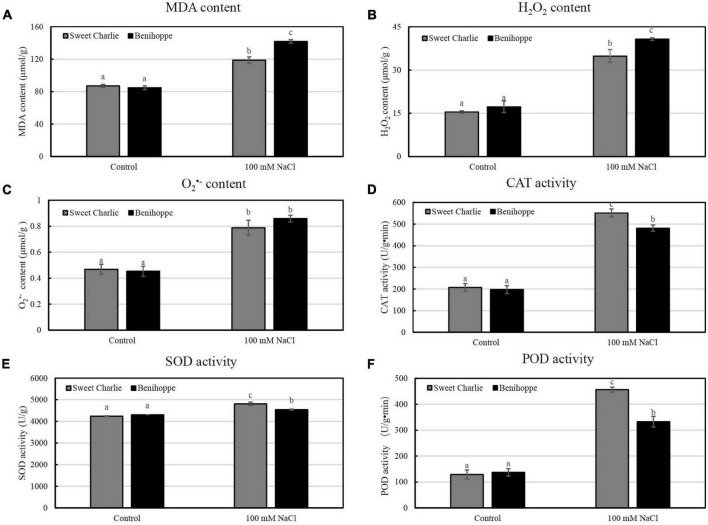
Physiological indices analysis of strawberry under salt stress. ‘Sweet Charlie’ and ‘Benihoppe’ plants with 6–7 leaves were treated with 0 mM (control) or 100 mM NaCl solution, respectively. After 10 days of treatment, the young leaves were collected for physiological indices determination. The content of MDA **(A)**, H_2_O_2_ **(B)**, and O_2_^•–^ **(C)** under control and salt stress treatment in strawberry leaves. The activities of CAT **(D)**, SOD **(E)**, and POD **(F)** under control and salt stress treatment in strawberry leaves. Data are means (±SD) of three independent experiments. Different letters indicate significant differences (*P* < 0.05).

To investigate the physiological changes in the two representative varieties of ‘Sweet Charlie’ and ‘Benihoppe’ under salt stress, strawberry plants at the 6–7 leaf stage, were treated with 100 mM NaCl for 10 days. Next, several physiological parameters were measured. Under normal conditions, the content of MDA in leaves did not differ between ‘Sweet Charlie’ and ‘Benihoppe.’ However, after salt stress, the content of MDA in ‘Benihoppe’ was higher than that of ‘Sweet Charlie’ (Figure 3A). Excessive accumulation of ROS is a consequence of salt stress and leads to membrane lipid peroxidation. MDA is a marker of membrane lipid peroxidation. In addition, the accumulation of hydrogen peroxide and superoxide anion were increased in both ‘Benihoppe’ and ‘Sweet Charlie’ under salt stress condition, with higher values in ‘Benihoppe’ compared to the levels in ‘Sweet Charlie’ (Figures 3B,C). The opposite trend was observed for SOD, POD, and CAT activities, with all three parameters significantly higher in ‘Sweet Charlie’ than in ‘Benihoppe’ under salt stress condition (Figures 3D–F). Thus, the results indicate greater salt sensitivity of ‘Benihoppe’ than ‘Sweet Charlie.’

### Transcriptomic profiles in ‘Benihoppe’ and ‘Sweet Charlie’ under salt stress

**FIGURE 4:**
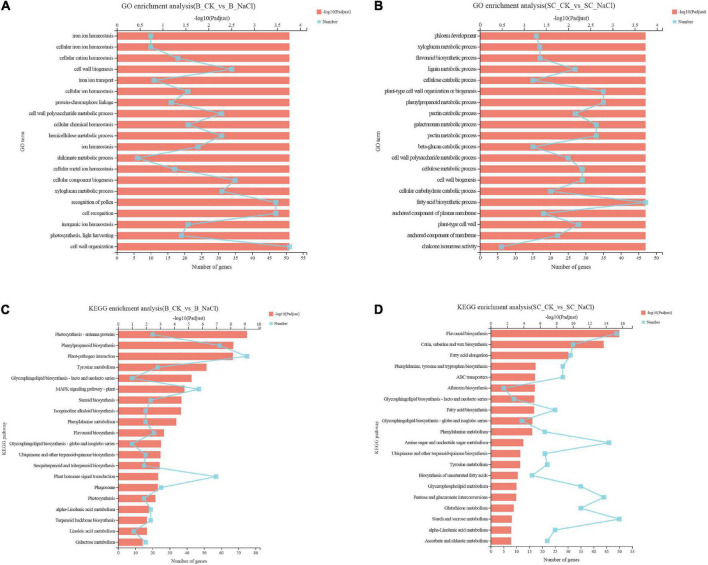
Gene Ontology enrichment analysis and KEGG pathway analysis of differentially expressed genes in strawberry leaves under salt stress. ‘Sweet Charlie’ and ‘Benihoppe’ plants with 6–7 leaves were treated with 0 mM (control) or 100 mM NaCl solution, respectively. After 12 h of treatment, the young leaves were collected for RNA-seq analysis. GO enrichment analysis and KEGG pathway analysis were performed to explore the functional significance of DEGs. Top 20 GO enrichment terms of differentially expressed genes in ‘Benihoppe’ **(A)** and ‘Sweet Charlie’ **(B)** under salt stress. Top 20 KEGG pathways of differentially expressed genes in ‘Benihoppe’ **(C)** and ‘Sweet Charlie’ **(D)** under salt stress.

The genetic and biochemical responses of ‘Benihoppe’ and ‘Sweet Charlie’ to salt stress were further explored using RNA-seq analysis. A summary of the sequencing data is presented in Supplementary Table 2. Principal component analysis (PCA) showed clear separations between each group (Supplementary Figure 1). In ‘Benihoppe,’ compared with normal condition, 3412 genes were differentially expressed under 100 mM NaCl treatment, with 1869 were upregulated and 1543 downregulated (Supplementary Figure 2 and Supplementary Table 3). In ‘Sweet Charlie,’ compared with normal condition, 5102 genes were differentially expressed under 100 mM NaCl treatment, with 4396 upregulated and 706 downregulated (Supplementary Figure 2 and Supplementary Table 4). GO and GO enrichment analysis were performed to explore the functional significance of DEGs. In ‘Benihoppe,’ 522 GO terms were annotated and 297 significant GO terms were obtained, with several ion homeostasis related terms ranked in the top 20 enriched terms (Figure 4A and Supplementary Table 5). In ‘Sweet Charlie,’ 572 GO terms were annotated and 339 significant GO terms were obtained, with a suite of cell wall-related terms ranked in the top 20 enriched terms (Figure 4B and Supplementary Table 5). KEGG pathway classification was performed to reveal the active biological pathways in strawberry in response to salt stress. In ‘Benihoppe,’ the DEGs mapped to 116 KEGG pathways, with 21 pathways were significantly enriched (Figure 4C and Supplementary Table 6). In ‘Sweet Charlie,’ the DEGs were mapped to 120 KEGG pathways, and 24 pathways were significantly enriched (Figure 4D and Supplementary Table 6). Only four KEGG enriched pathways were shared between ‘Benihoppe’ and ‘Sweet Charlie,’ including flavonoid biosynthesis, phenylpropanoid biosynthesis, phenylalanine metabolism and ubiquinone biosynthesis, and other terpenoid-quinone biosynthesis. This lack of substantial overlap indicates that ‘Sweet Charlie’ and ‘Benihoppe’ respond to salt stress using different pathways.

### Validation of ribonucleic acid-seq results by quantitative real-time polymerase chain reaction

**FIGURE 5:**
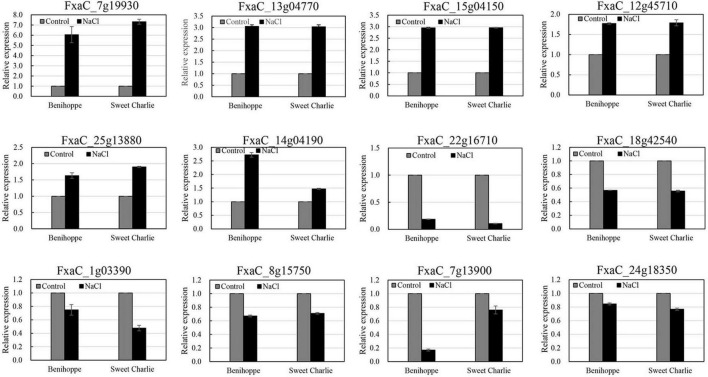
Verification of differentially expressed genes by qRT-PCR. ‘Sweet Charlie’ and ‘Benihoppe’ plants with 6–7 leaves were treated with 0 mM (control) or 100 mM NaCl solution, respectively. After 12 h of treatment, the young leaves were collected for RNA extraction. Twelve genes were randomly selected to validate the RNA-seq results *via* qRT-PCR. Data are means (±SD) of three independent experiments.

Twelve genes were randomly selected to validate the RNA-seq results *via* qRT-PCR, testing levels of six downregulated genes and six upregulated genes. The qRT-PCR analysis results showed similar gene expression trends as the observed changes in the RNA-seq data (Figure 5). These results confirmed the reliability of RNA-seq in this study.

### Metabolomic profiles in ‘Benihoppe’ and ‘Sweet Charlie’ under salt stress

**FIGURE 6:**
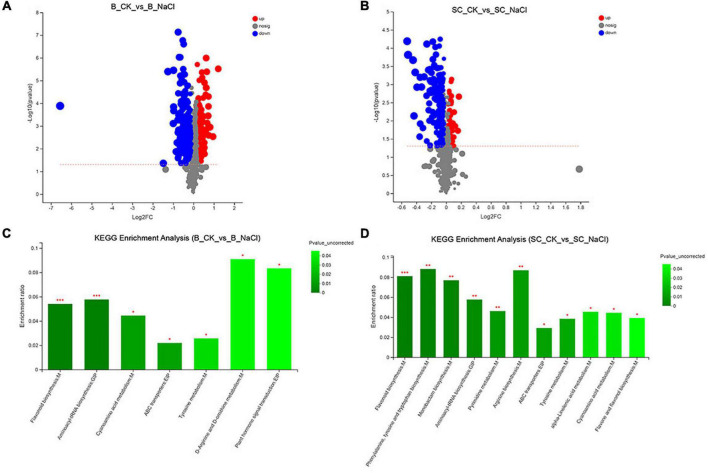
The statistics of differentially accumulated metabolites and KEGG enrichment under salt stress in strawberry. ‘Sweet Charlie’ and ‘Benihoppe’ plants with 6–7 leaves were treated with 0 mM (control) or 100 mM NaCl solution, respectively. After 12 h of treatment, the young leaves were collected for metabolite extraction. Non-targeted metabolomic analysis was performed to expose the metabolomic changes of ‘Benihoppe’ and ‘Sweet Charlie’ in response to salt stress. The volcano map of differentially accumulated metabolites in ‘Benihoppe’ **(A)** and ‘Sweet Charlie’ **(B)** leaves under salt stress. KEGG pathways of differentially accumulated metabolites in ‘Benihoppe’ **(C)** and ‘Sweet Charlie’ **(D)** under salt stress.

Non-targeted metabolomic analysis was performed to expose the metabolomic changes of ‘Benihoppe’ and ‘Sweet Charlie’ in response to salt stress. In ‘Benihoppe,’ 3105 metabolites and 209 DAMs were identified (80 upregulated and 129 downregulated, Figure 6A and Supplementary Table 7). The DAMs were mapped to 25 KEGG pathways, and seven pathways were significantly enriched (Figure 6C and Supplementary Table 8). In ‘Sweet Charlie,’ 3381 metabolites and 230 DAMs were identified (85 upregulated and 145 downregulated, Figure 6B and Supplementary Table 7), and the DAMs were mapped to 42 KEGG pathways with 11 pathways significantly enriched under salt stress (Figure 6D and Supplementary Table 8). Among the enriched KEGG pathways, five pathways were shared between ‘Benihoppe’ and ‘Sweet Charlie,’ including flavonoid biosynthesis, aminoacyl-tRNA biosynthesis, cyanoamino acid metabolism, adenosine triphosphate-binding cassette (ABC) transporters, and tyrosine metabolism.

### Ion homeostasis regulation under salt stress

**FIGURE 7:**
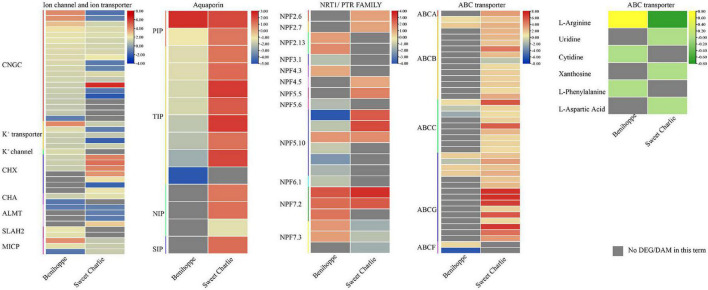
Differentially expressed genes related to ion channel and ion transporter, aquaporin, NRT1/PTR FAMILY, and ABC transporters, and differentially accumulated metabolites related to ABC transporters in ‘Benihoppe’ (left) and ‘Sweet Charlie’ (right) under salt stress. The relative expression levels as described by log_2_FC are represented by a color gradient from low (blue) to high (red). Under salt stress, the DEGs with log_2_FC > 0 represent upregulated expression, log_2_FC < 0 represents downregulated expression, and log_2_FC = 0 represents unchanged expression. Accumulation of metabolites as described by log_2_FC is represented by a color gradient from low (green) to high (yellow). Under salt stress, the DAMs with log_2_FC > 0 represent upregulated accumulation, log_2_FC < 0 represents downregulated accumulation, and log_2_FC = 0 represents unchanged accumulation. Gray squares indicate no differentially expressed genes in this term.

Ion channels and ion transporters play vital roles in maintaining ion homeostasis under salt stress. In this study, ion channels and ion transporters related genes showed differential response patterns to salt stress in ‘Benihoppe’ and ‘Sweet Charlie.’ In ‘Benihoppe,’ 19 cyclic nucleotide-gated ion channel (*CGNC*s), two S-type anion channel (*SLAH2*), one potassium channel encode gene, two mechanosensitive ion channel protein encoding genes, five potassium transporter encoding genes, three vacuolar cation/proton exchanger (*CHXs*), and one cation/H^+^ antiporter (*CHA*) gene were upregulated, while one *CNGC* gene, one mechanosensitive ion channel protein encoding gene, one aluminum-activated malate transporter (*ALMT*) gene, and one *CHA* gene were downregulated under salt stress. In ‘Sweet Charlie,’ 10 *CGNCs*, three mechanosensitive ion channel protein encoding genes, three potassium transporter encoding genes, one *ALMT* gene, six *CHX*s, and one *CHA* were upregulated, while six *CNGCs*, two potassium transporter encoding genes, three *ALMT*s, and one *CHX* gene were downregulated under salt stress (Figure 7 and Supplementary Table 9). Aquaporins play important roles in responsive to salt tolerance. In ‘Benihoppe,’ one plasma membrane intrinsic protein (*PIP*) gene was upregulated and one *PIP* and eight tonoplast intrinsic protein (*TIP*) genes were downregulated under salt stress. In ‘Sweet Charlie,’ seven *TIP*s, two *PIP*s, two nodulin-like intrinsic protein (*NIP*s), and one small and basic intrinsic proteins (*SIP*) gene were upregulated, while one NIP gene was downregulated (Figure 7 and Supplementary Table 9). NPF (NRT1/PTR FAMILY) belong to the large PTR (peptide transporter) family, and plant NPFs can identify a wide variety of substrates, including NO_3_^–^, NO_2_, Cl^–^, abscisic acid (ABA), auxin (IAA), and gibberellins (GAs) (Corratge-Faillie and Lacombe, 2017). Under salt stress, nine *NPF*s were upregulated, and eight *NPF*s were downregulated in ‘Benihoppe,’ with nine *NPF*s upregulated and three *NPF*s downregulated in ‘Sweet Charlie’ (Figure 7 and Supplementary Table 9). ABC transporters utilize the energy released from ATP hydrolysis to transport substrates and participate in the response to salt stress. Under salt stress, seven ABCs were upregulated and four ABCs were downregulated in ‘Benihoppe,’ while 38 ABCs were upregulated and one ABC was downregulated in ‘Sweet Charlie.’ In ‘Benihoppe,’ the differentially expressed *ABC*s belong to B, C, G, and F subfamilies, while in ‘Sweet Charlie,’ the differentially expressed *ABC*s belong to A, B, C, and G subfamilies. In ‘Benihoppe,’ three DAMs related to the ABC transporter pathway were identified, including L-Arginine, Cytidine, and L-Phenylalanine with L-Phenylalanine downregulated, and L-Arginine and Cytidine upregulated under salt stress. In ‘Sweet Charlie,’ four DAMs related to ABC transporter pathway were identified, including L-Arginine, Uridine, Xanthosine, and L-Aspartic Acid, with L-Arginine upregulated and Uridine, Xanthosine, and L-Aspartic Acid downregulated under salt stress (Figure 7 and Supplementary Table 9).

### Cell wall remodeling genes under salt stress

**FIGURE 8:**
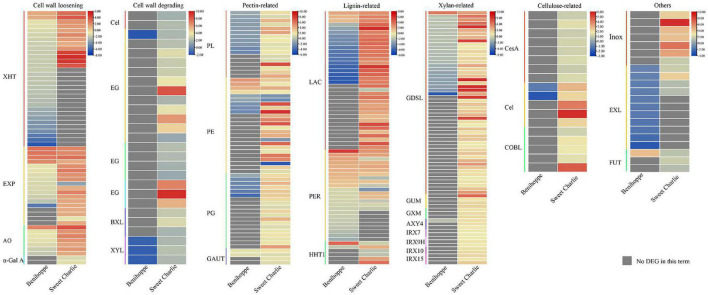
Differentially expressed genes related to cell wall remodeling in ‘Benihoppe’ (left) and ‘Sweet Charlie’ (right) under salt stress. The relative expression levels as described by log_2_FC are represented by a color gradient from low (blue) to high (red). Under salt stress, DEGs with log_2_FC > 0 represent upregulated expression, log_2_FC < 0 represents downregulated expression, and log_2_FC = 0 represents unchanged expression. Gray squares indicate no differentially expressed gene or differentially accumulated metabolite in this term.

In this study we identified multiple cell wall-related genes that exhibited differential expression in strawberry in response to salt stress. Xyloglucan endotransglucosylase/hydrolase (XHT) (Sharples et al., 2017), expansin (EXP) (Hepler et al., 2020), alpha-galactosidase (α-Gal A) (Chrost et al., 2007), and L-ascorbate oxidase (AO) (Green and Fry, 2005) participate in cell wall loosening. In‘Benihoppe,’ two *XHT*s, four *EXP*s, and one AO encoding genes were upregulated, while 29 *XHT*s, 10 *EXP*s, and six AO encoding genes were downregulated under salt stress. In ‘Sweet Charlie,’ 13 *XHT*s, 16 *EXP*s, and seven AO encoding genes were upregulated, while two *EXP*s and two α-Gal A encoding genes were downregulated under salt stress. Cellulase (Cel), endoglucanase, endo-beta-1,4-glucanase, endo-1,4-beta-glucanase, beta xylosidase (BXL), and beta-D-xylosidase (XYL) are well-known cell wall-degrading enzymes. In ‘Benihoppe,’ one endoglucanase encoding gene and three *XYL*s were downregulated under salt stress. In ‘Sweet Charlie,’ two *Cel*s, 12 endoglucanase encoding genes, four endo-beta-1,4-glucanase encoding genes, three endo-1,4-beta-glucanase encoding genes, three *BXL*s, and three *XYL*s were upregulated under salt stress (Figure 8 and Supplementary Table 10). Pectate lyase (PL), Pectinesterase (PE), and Polygalacturonase (PG) are pectin-degrading enzymes (Chen et al., 2018c,^2021^; Zhang et al., 2021a), and galacturonosyltransferase (GAUTs) are involved in pectin synthesis (Mohnen et al., 2012). In ‘Benihoppe,’ four *PE*s and two *GAUT*s were upregulated, while 11 *PL*s and five *PE*s were downregulated under salt stress. In ‘Sweet Charlie,’ 17 *PL*s, 20 *PE*s, 19 *PG*s, and four *GAUT*s were upregulated, and four *PE*s were downregulated under salt stress (Figure 8 and Supplementary Table 10). Cellulose synthase A (CesA), cellulose synthase-like (Csl), and COBRA-like (COBL) are involved in cellulose synthase (Li et al., 2019b; Daras et al., 2021). In ‘Benihoppe,’ two *Csl*s were downregulated under salt stress. In ‘Sweet Charlie,’ eight *CesA*s, five *Csl*s, and five *COBL*s were upregulated (Figure 8 and Supplementary Table 10). Laccase (LAC) and peroxidase (PER) are well-characterized lignin-related enzymes and omega-hydroxypalmitate *O*-feruloyl transferase (HHT1) participate in lignin monomer synthesis. In ‘Benihoppe,’ three *LACs*, 10 peroxidase encoding genes, and one omega-hydroxypalmitate *O*-feruloyl transferase encoding gene were upregulated, while 16 *LACs*, 14 peroxidase encoding genes, and one *HHT1* encoding gene were downregulated under salt stress. In ‘Sweet Charlie,’ 35 *LAC*s, nine peroxidase encoding genes, and three *HHT1* were upregulated, while one *LAC*, seven peroxidase encoding genes, and three *HHT1* encoding genes were downregulated under stress (Figure 8 and Supplementary Table 10). Glycosyltransferases, xylan alpha-glucuronosyltransferase (GUX), and DUF579 domain containing proteins IRX15-L are linked with xylan biosynthesis, and IRX7/9/10 are members of the glycosyltransferase family (Oikawa et al., 2010; Jensen et al., 2011, 2014). Glucuronoxylan 4-*O*-methyltransferase (GXM) involves xylan methylation, ALTERED XYLOGLUCAN 4 (AXY4) involves xylan acetylation, and GDSL esterase/lipase (GSDL) affects xylan deacetylation. In ‘Benihoppe,’ one *GDSL* was upregulated and one *AXY4* and 23 *GDSL*s were downregulated under salt stress. In ‘Sweet Charlie,’ four *GUX*s encoding genes, three *IRX15-L*, two *IRX7*, three *IRX9*, two *IRX10*, three GXM encoding genes, three *AXY4*s, and 52 *GDSL*s were upregulated under salt stress (Figure 8 and Supplementary Table 10). Inositol oxygenase is linked to the biosynthesis of nucleotide sugar precursors for cell-wall matrix polysaccharides (Kanter et al., 2005). In ‘Sweet Charlie,’ seven inositol oxygenase encoding genes were up regulated under salt stress (Figure 8 and Supplementary Table 10). The members of the PHI-1/EXO/EXL protein family participate in regulating secondary cell wall thickening and composition, and lignification (Sousa et al., 2020). Under salt stress, 11 *EXL3*s were downregulated in ‘Benihoppe,’ and five *EXL3*s were upregulated in ‘Sweet Charlie’ (Figure 8 and Supplementary Table 10). The galactoside 2-alpha-L-fucosyltransferase FUT1 adds a fucose residue to the 2-*O* position of terminal galactosyl residues on XyG side chains (Rocha et al., 2016). Under salt stress, one galactoside 2-alpha-L-fucosyltransferase encode gene was upregulated in ‘Benihoppe,’ and three galactoside 2-alpha-L-fucosyltransferase encoding genes were upregulated in ‘Sweet Charlie’ (Figure 8 and Supplementary Table 10).

### Flavonoid pathway under salt stress

**FIGURE 9:**
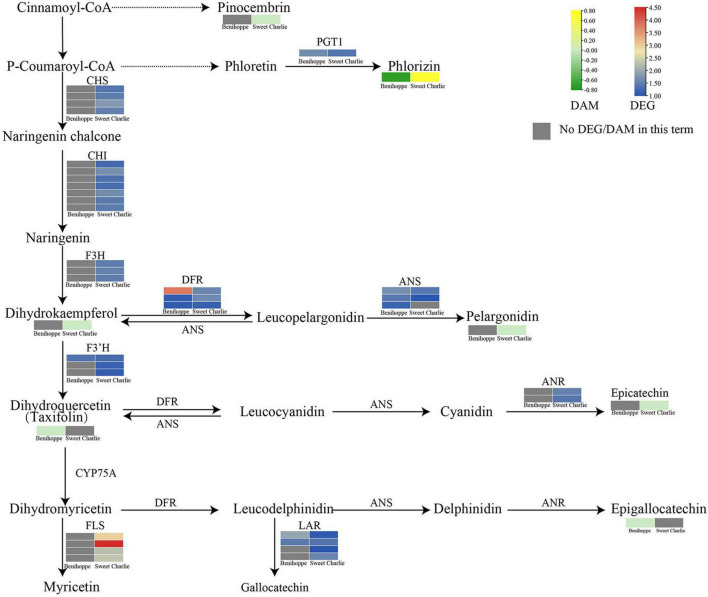
Differentially expressed genes and differentially accumulated metabolites related to flavonoid pathways in ‘Benihoppe’ (left) and ‘Sweet Charlie’ (right) under salt stress. The relative expression levels as described by log_2_FC are represented by a color gradient from low (blue) to high (red). Under salt stress, the DEGs with log_2_FC > 0 represent upregulated expression, log_2_FC < 0 represents downregulated expression, and log_2_FC = 0 represents unchanged expression. The accumulated of metabolites as described by log_2_FC are represented by a color gradient from low (green) to high (yellow). Under salt stress, the DAMs with log_2_FC > 0 represent upregulated accumulation, log_2_FC < 0 represents downregulated accumulation, and log_2_FC = 0 represents unchanged accumulation. Gray squares indicate no differentially expressed gene or differentially accumulated metabolite in this term.

Integrative analysis of transcriptomic and metabolomic data showed that the flavonoid pathway is involved in the salt stress response in strawberry. In ‘Benihoppe,’ the expression level of *FLS*s was reduced by salt stress, the expression of *F3’H*, *DFR*, *ANS*, *LAR*, and *PGT1* was induced by salt stress, and the content of epigallocatechin, taxifolin, and phloridzin decreased under salt stress. In ‘Sweet Charlie,’ salt stress induced expression of *CHS*, *CHI*, *F3H*, *FLS*, *F3’H*, *DFR*, *ANS*, *ANR*, *LAR*, and *PGT1;* while the levels of dihydrokaempferol, epicatechin, phloridzin, and pinocembrin were reduced by salt stress; and an increase in the content of pelargonidin was induced by salt stress (Figure 9 and Supplementary Table 11).

## Discussion

Salt stress disturbs cellular ionic homeostasis through the excessive accumulation of Na^+^ and Cl^–^. Plants have evolved efficient ion transport networks that enable them to maintain ion homeostasis, and maintaining a high K^+^/Na^+^ ratio in the cytosol is a key determinant of salinity tolerance (Almeida et al., 2017). CGNC, CHX, CHA, potassium channel, and potassium transporters play core roles in this process. The *Arabidopsis* genes *AtCNGC19* and *AtCNGC20* positively regulate plant salt tolerance, and *AtCNGC10* participates in K^+^ and Na^+^ uptake and long-distance transport (Guo et al., 2008). The CHX protein plays a critical role in maintaining K^+^ and Na^+^ homeostasis. In soybean, *GmCHX1* and *GmCHX20a* presented different expression patterns and opposite effects to salt tolerance (Jia et al., 2021). Chloride ions are a major factor contributing to ion toxicity and decreased production in strawberry (Suarez and Grieve, 2013; Devinder et al., 2019; Ferreira et al., 2019). To date, the most commonly reported gene families responsible for Cl^–^ transport are Cl^–^ channels (CLC), cation chloride channels (CCC), SLAH, ALMT, and NPF (Wu and Li, 2019). In this study, changes in the expression of different ion channel encoding genes and ion transporters were detected in ‘Benihoppe’ and ‘Sweet Charlie,’ with significant differences in Cl^–^ transport related genes. These results suggest different regulatory mechanisms of ion homeostasis between ‘Benihoppe’ and ‘Sweet Charlie’ (Figure 7 and Supplementary Table 9).

Aquaporins are a large family of transmembrane channel proteins that participate in the transport of water and nutrients. The aquaporins include five subfamilies of PIP, TIP, SIP, NIP, and X intrinsic proteins (XIP). Aquaporins help plants maintain water and ionic homeostasis and respond to salt stress. In wheat, *TaTIP4;1* serves as a positive regulator of salt tolerance by modulating water relations and the accumulation of Na^+^ (Wang et al., 2022). *ZxPIP1;3* conferred transgenic plants with salt tolerance by regulating water status and reducing ion toxicity (Li et al., 2021). *MaPIP1;1* enhanced banana salt tolerance *via* affecting the contents of Na^+^ and K^+^ (Xu et al., 2021). In this study, we found that *PIP* and *TIP*s participate in response to salt stress in ‘Benihoppe,’ while *TIP*, *PIP*s, *NIP*s, and *SIP* participate in response to salt stress in ‘Sweet Charlie.’ This suggests that different aquaporins are required for response to salt stress in different varieties, but *TIP* and *PIP* are the main aquaporins involved in response to salt stress in strawberry (Figure 7 and Supplementary Table 9).

The ABC transporters are one of the largest families of transporter proteins and are classified into eight subfamilies of ABCA-ABCI (ABCH transporters have not been identified in plants). ABC transporters are responsible for the transport of hormones, xenobiotics, amino acids, sugar, and ions, and are involved in plant growth and development regulation, nutrient uptake, response to biotic and abiotic stresses, and plant interactions with the environment. Recent findings have revealed roles of ABC transporters in response to salt stress. TsABCG11 improved salt tolerance in transgenic *Arabidopsis* seedlings (Chen et al., 2018b). In *Arabidopsis*, AtMRP5, a member of the ABC transporter family, can regulate *Arabidopsis* salt tolerance by altering K^+^ homeostasis (Lee et al., 2004), and AtABCG36/AtPDR8 improved salt resistance by reducing sodium content (Kim et al., 2010). In rice, OsABCG5 acts in the accumulation of essential and non-essential minerals and modulated rice salinity tolerance by affecting Na^+^/K^+^ homeostasis (Leng et al., 2014). ABCG and ABCB subfamilies tightly correlate with abiotic stress in plants (Linjun et al., 2019; Zhang et al., 2020). The ABCG subfamily is the largest group of the ABC transporter family and possesses the most complex function in response to abiotic stresses. In this study, ABCGs were more active in responses to salt stress, with one *ABCA*, 14 *ABCB*, nine *ABCC*, and 15 *ABCG* participating in salt stress responses in ‘Sweet Charlie,’ and two *ABCB*, three *ABCC*, four *ABCG*, and two *ABCF* participating in salt stress responses in ‘Benihoppe.’ Integrative analysis of transcriptomic and metabolomic data revealed that ABC transport plays an important role in response to salt stress in strawberry (Figure 7 and Supplementary Table 9).

Cell wall remodeling is a key aspect of plant acclimation to salt stress. Plant cell wall components are dynamically regulated in response to various environmental stresses. Salt stress can modify the deposition of cellulose, matrix, polysaccharides, and lignin. Under salt stress, the levels of cellulose and matrix polysaccharide were reduced but the content of lignin was increased in maize (Oliveira et al., 2020). In soybean, a salt-tolerant cultivar possessed a higher level of pectin than the sensitive line. Under salt stress, sodium ions affected pectin cross-links and disrupted microtubule stability to change cellulose deposition (An et al., 2014). Pectin usually presents in a highly methylesterified form. In *Populus tremula*, high methylated pectin within the cell wall was increased by salt stress (Muszyńska et al., 2014). Changes caused by salt stress results in cell wall remodeling to maintain cell wall integrity. Cell wall-loosening proteins such as *EXP*s and XTH play essential roles in response to salt stress. Overexpression of *EXP*s confers enhanced tolerance to salt stress in plants (Chen et al., 2017, 2018a; Jadamba et al., 2020). XTH positively regulated plant salt stress tolerance by regulating plant architecture (Ishida and Yokoyama, 2022). *COBL9* and *COBL7*, two COBRA-like family genes, are required for salinity tolerance and *COBL9* positively regulated root hair elongation and salinity tolerance in *Arabidopsis*; the rice counterparts of these genes, *OsBC1L1* and *OsBC1L8*, function redundantly in response to salinity stress (Li et al., 2022). Pectin plays an important role in adaptation to salt stress by regulating cell adhesion and tissue cohesion. In Arabidopsis, Pectin methylesterase 31 (*PME31*) positively modulated salt stress tolerance (Yan et al., 2018). Enhanced cell wall lignification is one of the main salinity tolerance strategies in the roots of halophytes. *LAC4* plays an important role in response to early stages of salt stress *via* affecting specialized protoxylem lignification in undifferentiated root tips (Barzegargolchini et al., 2017). In this study, more cell wall related genes were identified in ‘Sweet Charlie’ than in ‘Benihoppe,’ and those genes presented opposite expression patterns under salt stress. These results suggested that cell wall remodeling is a key driver of plant salt tolerance (Figure 8 and Supplementary Table 10).

In this study, we evaluated the salinity tolerance of 24 strawberry varieties and then clustered the varieties into three groups according to the salt damage indices. Physiological index analysis showed that ‘Benihoppe’ was more sensitive to salt stress than ‘Sweet Charlie,’ and this was consistent with the analysis of salt damage indices. Combined transcriptomic and metabolomic analysis showed that different pathways respond to salt stress in different varieties, and ABC transporters and cell wall remodeling play crucial roles in response to salt stress. Our results provide a foundation for the better understanding of the regulation mechanisms of the response of strawberry to salt stress.

## Data availability statement

The data presented in this study are deposited in the NCBI (National Center for Biotechnology Information) SRA repository, accession number PRJNA837123.

## Author contributions

SL, GW, YZ, and JS conceived this project and designed the research. SL, LC, and RS performed most of the experiments. JD, CZ, YG, HZ, LW, and YW participated in this work. SL and JS analyzed the data and wrote the manuscript. All authors discussed the manuscript, read, and approved the final manuscript.

## Footnotes

1
http://tophat.cbcb.umd.edu

2
http://deweylab.biostat.wisc.edu/rsem

3
http://www.bioconductor.org/packages/2.12/bioc/html/edgeR.html

4
https://github.com/tanghaibao/Goatools

5
http://kobas.cbi.pku.edu.cn/index.php

6
http://www.hmdb.ca/

7
https://metlin.scripps.edu/

8
https://cloud.majorbio.com

9
http://www.genome.jp/kegg/

## Supplementary material

Supplementary Figure 1 Principal component analysis (PCA) plots between individual samples for control and NaCl treatments.

Supplementary Figure 2 Volcano map of differentially expressed genes in ‘Benihoppe’ **(A)** and ‘Sweet Charlie’ **(B)** leaves under salt stress.
