# Supplementary figures and images for: Combined transcriptomic and metabolomic analysis reveals a role for adenosine triphosphate-binding cassette transporters and cell wall remodeling in response to salt stress in strawberry
Source: Front Plant Sci. 2022 Sep 6;13:996765. doi: 10.3389/fpls.2022.996765 (PMC9486094; doi:10.3389/fpls.2022.996765)

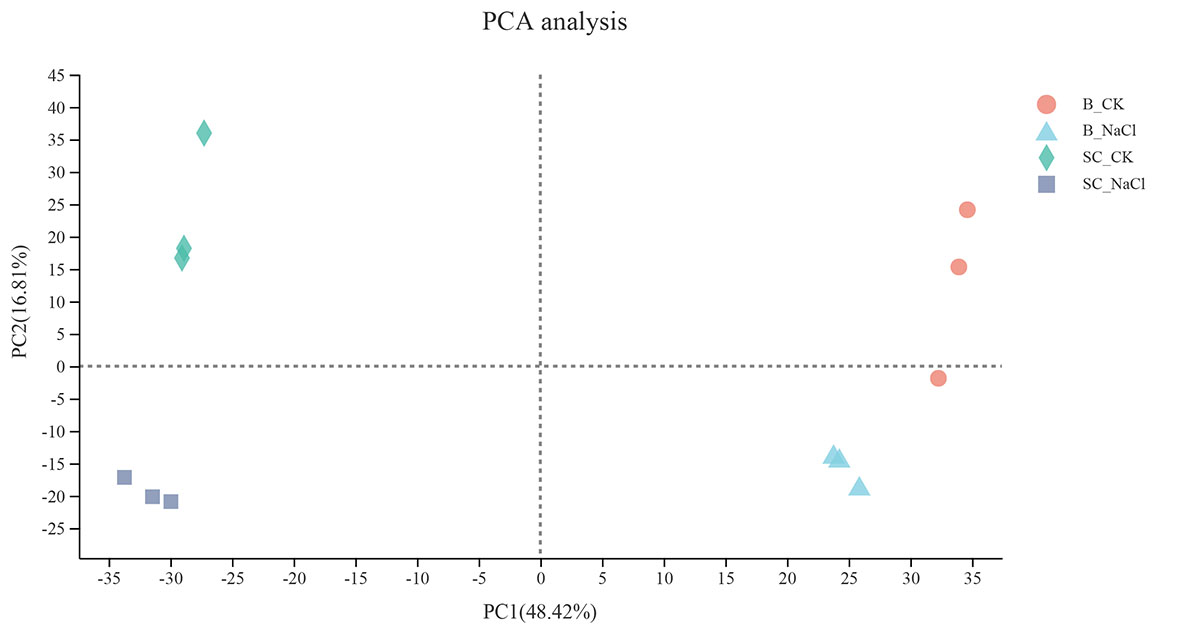

Supplement: Supplementary Figure 1 — Principal component analysis (PCA) plots between individual samples for control and NaCl treatments. [file Image_1.JPEG]

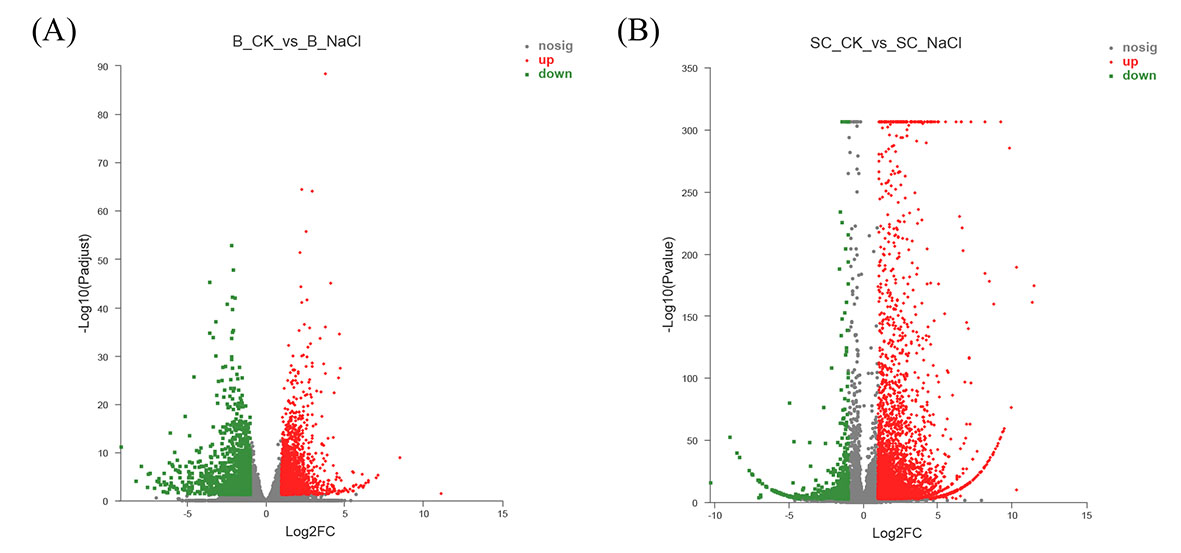

Supplement: Supplementary Figure 2 — Volcano map of differentially expressed genes in ‘Benihoppe’ (A) and ‘Sweet Charlie’ (B) leaves under salt stress. [file Image_2.JPEG]
